# Independent roles of country of birth and socioeconomic status in the occurrence of type 2 diabetes

**DOI:** 10.1186/1471-2458-13-1223

**Published:** 2013-12-23

**Authors:** Seyed Morteza Shamshirgaran, Louisa Jorm, Hilary Bambrick, Annemarie Hennessy

**Affiliations:** 1 School of Medicine, University of Western Sydney, Sydney, Australia; 2 Faculty of Health, Tabriz University of Medical Sciences, Tabriz, Iran; 3 Centre for Health Research, School of Medicine, University of Western Sydney, Sydney, Australia

**Keywords:** Type 2 diabetes, Country of birth, Ethnicity, Socioeconomic status

## Abstract

**Background:**

There is strong evidence based on previous studies that ethnicity and socioeconomic status are important determinants of diversity in the occurrence of diabetes. However, the independent roles of socioeconomic status, country of birth and lifestyle factors in the occurrence of type 2 diabetes have not been clearly identified. This study investigated the relationships between socioeconomic status, country of birth and type 2 diabetes in a large diverse sample of residents of New South Wales, Australia, and aged 45 years and over.

**Methods:**

The analysis used self-reported baseline questionnaire data from 266,848 participants in the 45 and Up Study. Educational attainment, work status and income were used as indicators of socioeconomic status. Logistic regression models were built to investigate associations between socioeconomic status, country of birth and type 2 diabetes.

**Results:**

The adjusted odds of type 2 diabetes were significantly higher for people born in many overseas countries, compared to Australian-born participants. Compared with participants who had a university degree or higher qualification, the adjusted odds ratio (OR) for diabetes was higher in all other educational categories. Diabetes was more prevalent in people who were retired, unemployed or engaged in other types of work, compared with people who were in paid work. The prevalence of diabetes was higher in people with lower incomes. Compared with people who earned more than $50,000, the adjusted OR for diabetes was 2.05 (95% CI 1.95-2.14) for people who had an income less than $20,000 per annum. The relationships between socioeconomic factors and country of birth and diabetes were attenuated slightly when all were included in the model. Addition of smoking, obesity and physical activity to the model had marked impacts on adjusted ORs for some countries of birth, but relationships between diabetes and all measures of socioeconomic status and country of birth remained strong and significant.

**Conclusions:**

Country of birth and socioeconomic status are independent predictors of type 2 diabetes. However, in this population, country of birth had a stronger association with type 2 diabetes.

## Background

Type 2 diabetes is a major health problem that is increasing at an alarming rate around the world. In 2010, there were 285 million people with diabetes globally. This number will increase to reach 439 million people during the next two decades, potentially leading to major medical, social, and economic problems [1]. Type 2 diabetes is one of the major health challenges for Australia, like other developed countries. It is estimated that 275 Australian adults develop type 2 diabetes every day [2]. Based on the results of the 2004–05 National Health Survey (which used self-report), 699,600 Australians had known diabetes [3]. However, the Ausdiab study in 1999–2000, which included measures of fasting blood glucose, estimated that 940,000 Australian adults (≥25 years old) had diabetes [4].

Australia is one of the most ethnically and culturally diverse countries in the world. In 2009, approximately one quarter (5.8 million people) of the Australian resident population (22 million people) was born overseas, of which more than half were from a non-English-speaking origin. The largest group of migrants were born in the United Kingdom (5.4% of Australia’s total population), followed by people born in New Zealand (2.4%). People born in Italy, China, and Vietnam were the next largest overseas-born group [5].

There is strong evidence based on previous studies around the world that ethnicity is an important determinant of diversity in the occurrence of diabetes [6]. Racial and ethnic disparities as a non-modifiable factor for diabetes emphasize the role of genetic and biomedical factors. However a wealth of epidemiologic research has demonstrated the importance of socioeconomic factors as a modifiable factor in the occurrence of type 2 diabetes [7-9]. A recent systematic review and meta analysis [10] concluded that in high-, middle- and low-income countries, the risk of type 2 diabetes was associated with low socioeconomic position as defined according to low educational level and occupation, although available data for middle- and low-income countries were limited. However, the independent roles of socioeconomic status, country of birth and life style factors in the occurrence of type 2 diabetes have not been clearly identified. Recently, some studies have reported that the role of socioeconomic status is stronger than that of ethnicity [11,12]. However these studies included only a limited range of ethnic groups. Therefore, this issue needs to be explored further in populations with heterogeneous racial, ethnic and socioeconomic compositions.

In this study, we explored the relationships between country of birth, socioeconomic status and type 2 diabetes in a large diverse population sample of residents of New South Wales (NSW), Australia, and aged 45 years and over.

## Methods

### Data source and study population

We used baseline questionnaire data from the 45 and Up Study, a large-scale collaborative cohort study of people aged 45 years and over in NSW, Australia’s largest state. The study includes 266,848 people aged 45 years and over, who were selected at random from the Medicare Australia (Australia’s national health insurance system) database. Its methods are described in detail elsewhere [13]. In brief, participants were randomly sampled from the database of Australia’s universal health insurance provider, Medicare Australia, which provides virtually complete coverage of the general population, including some temporary residents and refugees. The study over-sampled, by a factor of two, individuals aged 80 years and over and people resident in rural areas; all residents of remote areas were sampled [13]. Around 11% of the entire NSW population aged 45 years and over (approximately 18% of those approached) were included in the sample. Participants joined the Study by completing a baseline questionnaire between Jan 2006 and April 2009 and giving signed consent for follow-up and linkage of their information to a range of health databases. The Study’s baseline questionnaire captured information on a wide range of factors relating to healthy ageing, including disease risks, quality of life and other health outcomes, exposures such as life style factors, environmental determinants, socioeconomic status and demographic factors including country of birth (for more information see: http://www.45andup.org.au).

The 45 and Up Study received ethical approval from the University of NSW Human Research Ethics Committee for its collection of baseline data.

### Definitions, classification and exclusions

This analysis used self-reported data from the 45 and Up Study baseline questionnaire (available at https://www.saxinstitute.org.au/our-work/45-up-study/). Type 2 diabetes was defined on the basis of answers to the question ‘Has a doctor EVER told you that you have Diabetes?’ If YES “please cross the box and give your age when the condition was first found.” Participants were classified as having type 2 diabetes if they responded ‘yes’ and reported an age at diagnosis of ≥25 years.

Age was grouped into four categories: 45–55, 55–65, 65–75, and ≥75 years. Country of birth was classified according to the Australian Bureau of Statistics (ABS) Standard Australian Classification of Countries (SACC) [14], as follows: Australia, New Zealand, Rest of Oceania and Antarctica, United Kingdom, Germany, Netherlands, Italy, Greece, Rest of Europe, Egypt, Lebanon, Rest of the Middle East and North Africa, Vietnam, Philippines, China, India, Sri Lanka, Rest of Asia, Americas, and South Africa. Each of these groupings included 100 or more people with diabetes. People born in countries of Sub-Saharan Africa were excluded from the analysis because these comprised less than 100 people with diabetes.

Variables related to socio-economic status included educational qualification, work status, income and private health insurance. Educational qualification was classified as no formal qualification, school intermediate education, trade, apprenticeship, and university and higher education. Work status was categorized as in paid work, retired and other. Annual household income was categorized as more than $AU50,000, $AU20,000 to < $AU50,000, <$AU20,000 and not disclosed. Body Mass Index (BMI) was classified into four categories: underweight (BMI < 20 kg/m^2^), healthy weight (25 kg/m^2^ > BMI ≥ 20 kg/m^2^), overweight (30 kg/m^2^ > BMI ≥ 25 kg/m^2^) and obese (BMI ≥ 30 kg/m^2^). BMI values of less than nine and more than 50 were considered as invalid and these participants were excluded from analysis. Physical activity was defined as sessions per week including the sum of the total number of sessions spent walking and doing moderate activity and double the number of sessions of vigorous activity. This was based on the questions: “In the last week how many times have you walked continuously for at least 10 minutes for recreation or exercise or to get to or from places?”; “how many times did you do any vigorous physical activity that made you breathe harder or puff and pant?”; and “In the last week how many times did you do any other more moderate physical activity that you have not already mentioned?” Sessions of physical activity were grouped into four categories: None, ≤5, >5- ≤ 20, and more than 20 sessions per week. Where more than 200 sessions per week were reported, this was considered as invalid and the record was excluded from analysis.

Smoking status was classified into two categories: never being a regular smoker or ever being a regular smoker.

### Statistical analysis

Analysis was performed using the STATA statistical package Version 11. Univariate analyses were used to describe the general characteristics of the study population. Multivariable logistic regression models with self-reported diabetes as the dependent variable were used to estimate odds ratios (ORs) with 95% confidence intervals (CI). Three models were built: adjusted for age and sex only; adjusted, for age, sex, country of birth and measures of socioeconomic status (household income, educational qualification, work status); and adjusted for age, sex, country of birth, measures of socioeconomic status and lifestyle-related factors (BMI, physical activity, and smoking). Significance was considered at the 5% level.

## Results

After excluding 333 volunteers, those who were diagnosed with diabetes at less than 25 years of age (470), those who had unknown country of birth (179) or insufficiently described country of birth (2545), 262,233 participants were included in the subsequent analysis. Of these, 23,112 (8.81%) reported having diabetes. Compared with other 45 and Up Study participants, people with diabetes were older and more likely to be male. They were significantly more likely to be overweight or obese, to report fewer weekly sessions of physical activity and to have smoked (Table 1).

**Table 1:** Characteristics of the study population according to self-reported type 2 diabetes*

	**Type 2 diabetes**			
**Variables**	**No**		**Yes**	
	**No**	**%**	**No**	**%**
**Age (years)**				
45-55	73236	30.6	3330	14.4
55-65	77765	32.5	6771	29.3
65-75	49924	20.9	7096	30.7
≥75	38280	16.0	5912	25.6
**Sex**				
Male	108671	45.4	13099	56.7
Female	130549	54.6	10013	43.3
**Body mass index (BMI)**^**†**^				
Underweight	9656	4.50	389	1.9
Healthy weight	78509	35.4	3984	18.9
Overweight	88113	43.0	7860	37.3
Obese	45364	20.5	8822	41.9
**Physical activity**				
>20 sessions/week	31833	13.6	2276	10.2
>5-≤20 sessions/week	144548	61.7	12366	55.3
≤5 sessions/week	46132	19.7	5628	25.2
None	11720	5.0	2083	9.3
**Ever smoker**				
No	136326	57.0	11443	49.5
Yes	102813	43.0	11656	50.5
**Total**	**239220**	**100**	**23112**	**100**

^*^Numbers obtained by adding categories together for each variable may not add to total due to missing data.

^†^Underweight: BMI < 20; healthy weight: 25 > BMI ≥ 20; overweight: 30 > BMI ≥ 25; obese: BMI ≥ 30.

Diabetes was more prevalent in migrant groups than in Australian-born people. The prevalence of type 2 diabetes was 8.6% among Australian-born participants and 12.7% in overseas-born participants as a group. For men, prevalence was higher among those from Sri Lanka (28.0%), Lebanon (21.9%), Philippines (20.2%), India (19.8%), Egypt (18.7%), rest of the Middle East and North Africa (19.5%), Italy (17.6%) and rest of Oceania and Antarctica (16.8%). For women, prevalence was higher among those from Sri Lanka (15.9%), Lebanon (14.7%), Philippines (13.8%), and India (13.3%).

After adjustment for age and sex, the odds of reporting type 2 diabetes was elevated for people born in other countries of Oceania and Antarctica (OR 2.19 95% CI 1.81-2.65), Lebanon (2.73 95% CI 2.24-3.31), rest of the Middle East and North Africa (2.20 95% CI 1.83-2.64), the Philippines (2.64 95% CI 2.26-3.09), India (2.19 95% CI 1.87-2.57) and Sri Lanka (3.42 95% CI 2.77-4.22) (Table 2).

**Table 2:** Country of birth and type 2 diabetes among 45 and up study participants

	**Diabetes**									
**Country/region of birth**	**No**		**Yes**		OR (1)^*^	**95% CI**	**OR (2)**^**†**^	**95% CI**	**OR (3)**^**‡**^	**95% CI**
	**N**	**%**	**N**	**%**						
**Australia**	181,820	91.6	16,735	8.4	1.00	-	1.00	-	1.00	-
**New Zealand**	4,699	93.7	317	6.3	0.79	0.70-0.89	0.84	0.74-0.94	0.87	0.77-0.99
**Rest of Oceania & Antarctica**	754	85.2	131	14.8	2.19	1.81-2.65	2.21	1.81-2.70	2.18	1.74-2.74
**United Kingdom**	22,746	91.3	2,162	8.7	0.94	0.89-0.98	0.97	0.92-1.02	1.02	0.97--.08
**Germany**	2,506	90.4	267	9.6	1.02	0.89-1.15	1.00	0.87-1.14	1.00	0.87-1.16
**Netherland**	2,333	89.1	284	10.9	1.08	0.95-1.23	1.07	0.93-1.22	1.05	0.91-1.21
**Italy**	1,795	84.7	324	15.3	1.58	1.40-1.78	1.40	1.22-1.59	1.26	1.09-1.46
**Greece**	719	86.0	117	14.0	1.48	1.21-1.80	1.20	0.97-1.49	1.13	0.90-1.42
**Rest of Europe**	7,263	89.1	885	10.9	1.16	1.07-1.24	1.11	1.02-1.19	1.10	1.01-1.19
**Egypt**	542	83.8	105	16.2	1.88	1.52-2.32	1.78	1.41-2.24	1.53	1.19-1.97
**Lebanon**	560	81.0	131	19.0	2.73	2.24-3.31	2.12	1.72-2.60	1.97	1.57-2.47
**Rest of the Middle East & North Africa**	755	84.0	143	16.0	2.20	1.83-2.64	1.86	1.52-2.27	1.58	1.26-1.97
**Vietnam**	722	87.0	108	13.0	1.93	1.57-2.37	1.50	1.21-1.86	2.84	2.26-3.57
**Philippines**	1,057	84.4	196	15.6	2.64	2.26-3.09	2.97	2.52-3.51	4.16	3.47-4.99
**China**	1,691	90.8	172	9.2	1.17	1.00-1.38	1.10	0.931.30	1.80	1.51-2.16
**India**	931	82.9	192	17.1	2.19	1.87-2.57	2.63	2.22-3.10	3.26	2.74-3.90
**Sri Lanka**	405	77.3	119	22.7	3.42	2.77-4.22	4.07	3.26-5.08	5.73	4.52-7.26
**Rest of Asia**	3,122	90.3	335	9.7	1.39	1.24-1.56	1.42	1.26-1.60	2.17	1.91-2.46
**Americas**	2,786	92.4	224	7.6	0.95	0.83-1.10	1.08	0.94-1.25	1.16	0.99-1.35
**South Africa**	1,329	92.7	104	7.3	0.88	0.72-1.08	1.07	0.87-1.32	1.16	0.92-1.45

^*^OR(1): adjusted for age and sex.

^**†**^OR(2): adjusted for age, sex, country of birth, work status, household income, educational qualification.

^**‡**^OR(3): adjusted for age, sex, country of birth, work status, household income, educational qualification, BMI, smoking, sessions of physical activity.

Compared with participants who had a University degree or higher qualification, the age and sex-adjusted OR of diabetes was higher in all other educational categories, ranging from 1.27 (95% CI 1.21-1.32) for people with a trade certificate or diploma to 1.63 (95% CI 1.55-1.72) for people with no qualifications. Compared with people whose annual household income was more than $50,000, the age- and sex-adjusted OR of diabetes was higher for those on lower incomes; 1.37 (95% CI 1.31-1.43) for those between $20,000 and $50,000, and 2.05 (95% CI: 1.95-2.14) for those on less than $20,000 per annum. Compared with people who were in paid employment, the age and sex-adjusted OR for diabetes was higher in people who were retired (1.22 95% CI 1.16-1.29), or who were unemployed or involved in other type of work (1.17 95% CI 1.12-1.23) (Table 3).

**Table 3:** Socioeconomic factors and type 2 diabetes among 45 and up study participants

	**Diabetes**									
**Socioeconomic factor**	**No**		**Yes**		OR (1)^*^	**95% CI**	**OR (2)**^**†**^	**95% CI**	**OR (3)**^**‡**^	**95% CI**
	**N**	**%**	**N**	**%**						
**Educational qualification**										
University and higher	56,919	94.2	3,538	5.8	1.00	-	1.00	-	1.00	-
Trade, apprenticeship	76,569	91.6	6,984	8.4	1.27	1.21-1.32	1.32	1.26-1.38	1.17	1.11-1.22
School intermediate	75,773	90.7	7,790	9.3	1.13	1.08-1.18	1.19	1.14-1.25	1.79	1.03-1.13
No formal qualification	26,387	86.0	4,278	14.0	1.63	1.55-1.72	1.71	1.62-1.80	1.36	1.30-1.46
**Annual household income**										
>$AU50,000	84,763	94.9	4,548	5.1	1.00	-	1.00	-	1.00	-
$AU20,000-<$AU50,000	59,117	91.1	5,801	8.9	1.37	1.31-1.43	1.34	1.28-1.40	1.32	1.26-1.38
<$AU20,000	44,001	85.5	7,472	14.5	2.05	1.95-2.14	1.95	1.86-2.05	1.79	1.70-1.88
Did not disclose	39,140	90.7	4,005	9.3	1.46	1.39-1.54	1.42	1.35-1.49	1.36	1.29-1.43
**Work status**										
In paid work	77,525	95.0	4,051	5.0	1.00	-	1.00	-	1.00	-
Retired	82,569	87.7	11,565	12.3	1.22	1.16-1.29	1.26	1.20-1.33	1.25	1.18-1.32
Other	77,252	91.5	7,203	8.5	1.17	1.12-1.23	1.20	1.14-1.25	1.21	1.15-1.26

^*^OR(1): adjusted for age and sex.

^**†**^OR(2): adjusted for age, sex, country of birth, work status, household income, educational qualification.

^‡^OR(3): adjusted for age, sex, country of birth, work status, household income, educational qualification, BMI, smoking, sessions of physical activity.

The relationships between diabetes, country of birth and socioeconomic factors were attenuated slightly when all variables were included in the model. Addition of smoking, obesity and physical activity to the model along with country of birth and socioeconomic status measures reduced the adjusted ORs slightly for some countries, but increased ORs for countries including Vietnam (2.84 95% CI 2.26-3.57), the Philippines (4.16 95% CI 3.47-4.99), India (3.26 95% CI 2.74-3.90), Sri Lanka (5.73 95% CI 4.52-7.26), and rest of Asia (2.17 95% CI 1.91-2.46). Relationships between diabetes and country of birth after adjusting for all other covariates of interest including socioeconomic status remained strong and significant. Relationships between diabetes and socioeconomic status in all three models remained significant with minimal changes.

## Discussion

Our study used data from a large study with a diverse population base to examine the independent roles of ethnicity and socioeconomic status in the occurrence of type 2 diabetes. The results showed that diabetes was more prevalent in people born in many overseas countries, in particular Asian and the Middle Eastern countries, compared to those born in Australia. This is consistent with previous studies both in Australia [15,16] and other countries [6,17-21] that have compared rates of diabetes in migrants with those in native-born populations. In a study from Canada, type 2 diabetes was reported to be higher in South Asian migrants compared to Canadian- born population [21]. A study in the United States of America that compared the prevalence of type 2 diabetes between Asian Americans and the white population over the period 1997–2007 found that Asian Americans had 30-50% higher prevalence of type 2 diabetes compare to their non-Hispanic white counterparts, despite having lower BMI. People born in India had the highest odds of prevalent type 2 diabetes, followed by those born in the Philippines and China [22]. In the UK, the prevalence of type 2 diabetes among South Asian migrants is more than five times greater than in native British people [18]. Ethnic variations in the prevalence of type 2 diabetes are hypothesised to be caused by differences in genetic susceptibility as well as in socioeconomic and lifestyle risk factors [6,18,23].

Our finding that the prevalence of type 2 diabetes is inversely related to socioeconomic status also confirmed those of previous studies [7-9] and systematic reviews [10]. Increased risk in people of low socioeconomic status is thought to be mediated through lifestyle and environmental risk factors [24,25].

The unique contribution of this study was to clarify the independent contributions of country of birth and socioeconomic status to the risk of type 2 diabetes, using a large sample size drawn from an ethnically diverse population. We found that variations in prevalence according to country of birth were much larger than variations according to socioeconomic status, and that these disparities remained, and sometimes increased, after adjustment for socioeconomic factors. This contrasts with a population-based study in Boston, USA, that concluded that the role of socioeconomic status is stronger than ethnicity and emphasized that socioeconomic status, as a modifiable determinant should receive greater attention than ethnicity, a non-modifiable factor [11]. This discrepancy may be explained by the much greater ethnic diversity present in our study population. The Boston study, for example, included no migrants from Asian or Middle Eastern countries.

In general after adjustment for lifestyle factors, we found that associations between diabetes and both country of birth and measures of socioeconomic status were attenuated but remained statistically significant. This suggests that these factors alone did not explain the variation in diabetes prevalence and that other, unmeasured factors, such as genetic susceptibility, diet, environmental factors and gene-environment interactions play a role [6,18,23].

Particular strengths of this study were its very large and diverse sample, and the availability of detailed individual data on a range of measures of socioeconomic status including education, income and job status, as well as lifestyle factors. However, we did not have detailed information about race and ethnicity, so could not explore the potential roles of genetic, cultural and environmental factors in explaining differences according to country of birth.

Diabetes status, as well as all predictor variables, was ascertained using a self-report method [26]. There is no reason to believe however, that self-reported diabetes would be more accurate for people born overseas or of lower socioeconomic status; in fact any misclassification is likely to have biased the observed associations towards the null.

## Conclusion

In conclusion, our findings emphasise the magnitude of ethnic variations in the prevalence of type 2 diabetes, which are independent of socioeconomic status and key lifestyle factors including obesity, smoking and physical activity. In particular, the risk of diabetes was increased three-fold or more for people born in the Philippines, India and Sri Lanka. Our study emphasises the importance of targeted programs for early intervention and diabetes management in people from high-risk ethnic backgrounds, and of further research to better characterise gene-environment interactions that might be amenable to modification through personalised medicine.

## Competing interests

The authors declare that they have no competing interests.

## Authors’ contributions

All authors were involved in design of the protocol and preparation of the Human Research Ethics Committee application. MSH was responsible for data analysis and prepared of drafts of the manuscript. LJ and HB and AH supervised and supported data analysis and contributed to all drafts of the manuscript, including the final one. All authors read and approved the final manuscript.

## Pre-publication history

The pre-publication history for this paper can be accessed here:

http://www.biomedcentral.com/1471-2458/13/1223/prepub
